# Ascorbate Enhances the Cytotoxicity of the Manganese–Curcumin–Triphenylphosphonium Complex in Human Cancer Cells

**DOI:** 10.3390/ph19101549

**Published:** 2026-09-30

**Authors:** Bogdan Alexandru Stoica, Victor Alexandru Constantinescu, Anton Knieling, Liliana Georgeta Foia, Laura Stoica, Bogdan Minea, Ioan-Adrian Ciureanu, Carmen Ecaterina Leferman, Oana Olariu, Cezar Ilie Foia, Andreea-Laura Antohi, Alin Dumitru Ciubotaru, Tudor Petreus

**Affiliations:** 1 Grigore T. Popa University of Medicine and Pharmacy, Strada Universitatii 16, 700115 Iasi, Romania; bogdan.stoica@umfiasi.ro (B.A.S.); victor.constantinescu@umfiasi.ro (V.A.C.); anton.knieling@umfiasi.ro (A.K.); georgeta.foia@umfiasi.ro (L.G.F.); laura.stoica@umfiasi.ro (L.S.); bogdan-minea@umfiasi.ro (B.M.); adrian.ciureanu@umfiasi.ro (I.-A.C.); carmen-ecaterina.leferman@umfiasi.ro (C.E.L.); oana-olariu@umfiasi.ro (O.O.); foia.cezar-ilie@d.umfiasi.ro (C.I.F.); mg-rom-33303@students.umfiasi.ro (A.-L.A.); tudor.petreus@umfiasi.ro (T.P.); 2 NETRI, 76 Rue Georges Gouy, 69007 Lyon, France

**Keywords:** vitamin C, curcumin, manganese complexes, triphenylphosphonium, reactive oxygen species, hydrogen peroxide, mitochondrial membrane potential, drug synergy

## Abstract

**Background/Objectives**: Manganese–Curcumin–Triphenylphosphonium (Mn-C-TPP), a complex designed for mitochondrial targeting, was investigated with ascorbate to assess cytotoxicity and associated cellular responses. We hypothesized that Mn-C-TPP would amplify ascorbate-driven oxidative stress and produce synergistic cytotoxicity in susceptible cancer cell lines. **Methods**: An inverse, control-normalized SYTOX-derived response index (vehicle = 100; staurosporine = 0) was determined after 72 h in HCT116, A549, HepG2, and normal dermal human fibroblasts (NDHFs). H_2_O_2_-associated luminescence (ROS-Glo), caspase-3/7 activation, and mitochondrial membrane potential were assessed in selected cancer cell lines. Drug interactions were analyzed using SynergyFinder 3.0 (ZIP model). **Results**: Mn-C-TPP alone reduced the SYTOX-derived response index in HCT116 and HepG2, whereas A549 and NDHF showed smaller changes. Ascorbate enhanced cytotoxicity at selected combinations. In HCT116, 3 µM Mn-C-TPP plus 100 µM ascorbate decreased the SYTOX-derived response index toward the staurosporine reference. HepG2 responded markedly to 3–7 µM Mn-C-TPP with 100–300 µM ascorbate, whereas pronounced A549 responses occurred mainly at 15–30 µM Mn-C-TPP with 100–300 µM ascorbate. Mean ZIP scores were approximately 32, 54, 6, and 10 for HCT116, HepG2, A549, and NDHF, respectively. The NDHF response index remained close to the vehicle reference at lower combination doses but decreased at higher doses. Cellular assays showed increased H_2_O_2_-associated luminescence with Mn-C-TPP alone and higher mean signals with selected combinations than with ascorbate alone, together with caspase-3/7 activation and mitochondrial depolarization. **Conclusions**: These findings demonstrate cell-line- and concentration-dependent cytotoxicity and support further investigation of the proposed mitochondrial redox mechanism.

## 1. Introduction

Advances in targeted therapy and immunotherapy have markedly improved the therapeutic landscape for cancer. Nevertheless, during chemotherapy, malignant cells may undergo adaptive changes that lead to the development of mechanisms of multidrug resistance (MDR), a phenomenon that substantially diminishes therapeutic efficacy and contributes to poor clinical outcomes [1,2]. Addressing this challenge requires the development of strategies capable of eliminating tumor cells while preserving normal tissue integrity [3]. A particularly promising direction involves targeting defined vulnerabilities in cancer cell metabolism and redox homeostasis to induce selective cytotoxicity, thereby providing a way to circumvent resistance mechanisms and enhance long-term therapeutic success [4,5].

Many cancer cell types exhibit elevated basal levels of reactive oxygen species (ROS) relative to their normal counterparts, a consequence of increased metabolic activity, mitochondrial dysfunction, and oncogene-driven signaling. This pro-oxidative state is not uniform across all malignancies but is a common feature in tumors with high proliferative rates or altered metabolic pathways [6,7]. Cancer cells adapt to persistent oxidative stress by upregulating antioxidant defense systems. However, when ROS production exceeds the neutralizing capacity of these defenses, it can cause irreversible damage to essential cellular structures and activate programmed cell death pathways. This susceptibility has informed therapeutic strategies designed to induce oxidative stress—either by further increasing ROS levels or by impairing antioxidant systems in tumor cells—thereby surpassing a cytotoxic oxidative threshold [8,9,10]. Differences in redox homeostasis and antioxidant defenses between malignant and non-malignant cells may influence their susceptibility to these interventions, creating a potential therapeutic window [11,12].

In this context, redox-active compounds designed to elevate ROS in cancer cells have been investigated as a potential means to overcome MDR and improve therapeutic selectivity [13,14,15]. The pro-oxidant therapeutic strategy has been investigated in malignancies with intrinsically high oxidative stress and accelerated metabolism, such as certain leukemias, aggressive breast cancers, and melanoma [16,17,18]. Agents act either by directly promoting ROS generation—through mitochondrial electron transport chain interference (e.g., arsenic trioxide) [19], redox cycling (e.g., β-lapachone, anthracyclines such as doxorubicin) [20,21], or iron-mediated Fenton chemistry [22]—or by disabling antioxidant defenses, such as glutathione depletion (e.g., phenethyl isothiocyanate, buthionine sulfoximine) [23,24] and inhibition of antioxidant enzymes (e.g., 2-methoxyestradiol for superoxide dismutase (SOD), PX-12 for thioredoxin) [25,26]. By targeting tumor cells already close to their oxidative limit, these interventions can induce irreversible oxidative damage to proteins, lipids, and nucleic acids, activating apoptosis or necrosis [9]. Furthermore, combination regimens that pair ROS-generating agents with inhibitors of antioxidant systems have demonstrated synergistic efficacy and the potential to overcome redox adaptation-mediated drug resistance, underscoring the therapeutic value of precisely modulating tumor redox balance for selective cytotoxicity [27,28].

Mitochondria play a central role in maintaining cellular redox balance and regulating cell death. This has led to the emergence of compounds that specifically target mitochondrial function, known as mitocans, which have shown considerable promise in cancer therapy [29,30]. These agents can induce tumor cell apoptosis by enhancing mitochondrial ROS production, disrupting membrane integrity, or impairing respiration. A prominent class within this group are triphenylphosphonium (TPP^+^)-conjugated compounds, which can preferentially accumulate in the hyperpolarized mitochondria of cancer cells, potentially increasing their selective toxicity toward malignant tissue [31,32].

Mn-C-TPP is a compound designed for mitochondrial targeting, composed of a manganese–curcumin complex linked to a triphenylphosphonium (TPP^+^) cation. This design integrates the redox-active properties of manganese with the anticancer potential of curcumin, while the TPP^+^ moiety is intended to promote mitochondrial accumulation. Previous work reported greater antiproliferative activity of a TPP–manganese–curcumin complex against cancer cells compared with curcumin alone [33]. However, whether combining Mn-C-TPP with ascorbate can enhance cytotoxicity in cancer cells while limiting toxicity toward non-malignant cells remains to be established. This question provides the rationale for investigating the combined treatment.

Ascorbic acid (vitamin C) acts as an antioxidant at physiological levels, but at high concentrations it can act as a pro-oxidant, generating hydrogen peroxide (H_2_O_2_) through auto-oxidation, particularly in the presence of catalytic metal ions [34,35]. Some cancer cells have a reduced capacity to metabolize H_2_O_2_ associated with lower catalase activity, increasing their susceptibility to peroxide-induced oxidative damage [36]. As a monotherapy, vitamin C may require high concentrations to exert cytotoxic effects, so combining it with other treatments is a promising strategy [37]. Notably, high-dose ascorbate has demonstrated synergy with certain chemotherapies and radiation, an effect attributed to augmented ROS generation and tumor oxidative injury [38].

Redox-active manganese catalysts—particularly Mn porphyrins—can catalyze ascorbate oxidation and markedly enhance H_2_O_2_-dependent cytotoxicity, producing synergy in multiple cancer models [39,40,41,42]. Unlike the previously investigated Mn porphyrin/ascorbate systems, Mn-C-TPP combines a manganese–curcumin complex with a mitochondria-targeting TPP^+^ moiety. We therefore hypothesized that combining Mn-C-TPP with ascorbate could enhance cytotoxicity in cancer cells, potentially through effects on mitochondrial function and redox homeostasis [31,33].

To explore these hypotheses, a panel of human cancer cell lines of diverse tissue origin (colorectal, lung, and liver) was studied alongside normal dermal fibroblasts as a non-malignant control. Cell viability index and drug interactions were evaluated following 72 h exposure to Mn-C-TPP and ascorbate, alone or in combination. H_2_O_2_-associated luminescence was compared among vehicle, ascorbate-only, Mn-C-TPP-only, and combination conditions in HCT116 and HepG2 cells. Caspase-3/7 activation and mitochondrial membrane potential (ΔΨm) were assessed in selected cancer cell lines to further characterize cellular responses to treatment.

## 2. Results

### 2.1. SYTOX Green-Based Assessment of Cell Viability After 72 h Exposure

Across HCT116, A549 and HepG2 cells, 72 h co-treatment with Mn-C-TPP and ascorbate at 100–300 µM produced changes consistent with reduced viability in a cell line- and concentration-dependent manner, with pronounced effects at Mn-C-TPP concentrations of 15–30 µM. Ascorbate at 50 µM in combination with Mn-C-TPP enhanced cytotoxicity in HCT116 and HepG2 cells, whereas 300 µM ascorbate with higher Mn-C-TPP doses led to low response index values in all tumor lines. In contrast, NDHFs showed little evidence of viability loss under most conditions, with decreases in the response index observed mainly at the highest combinations of Mn-C-TPP and ascorbate.

**Figure 1:**
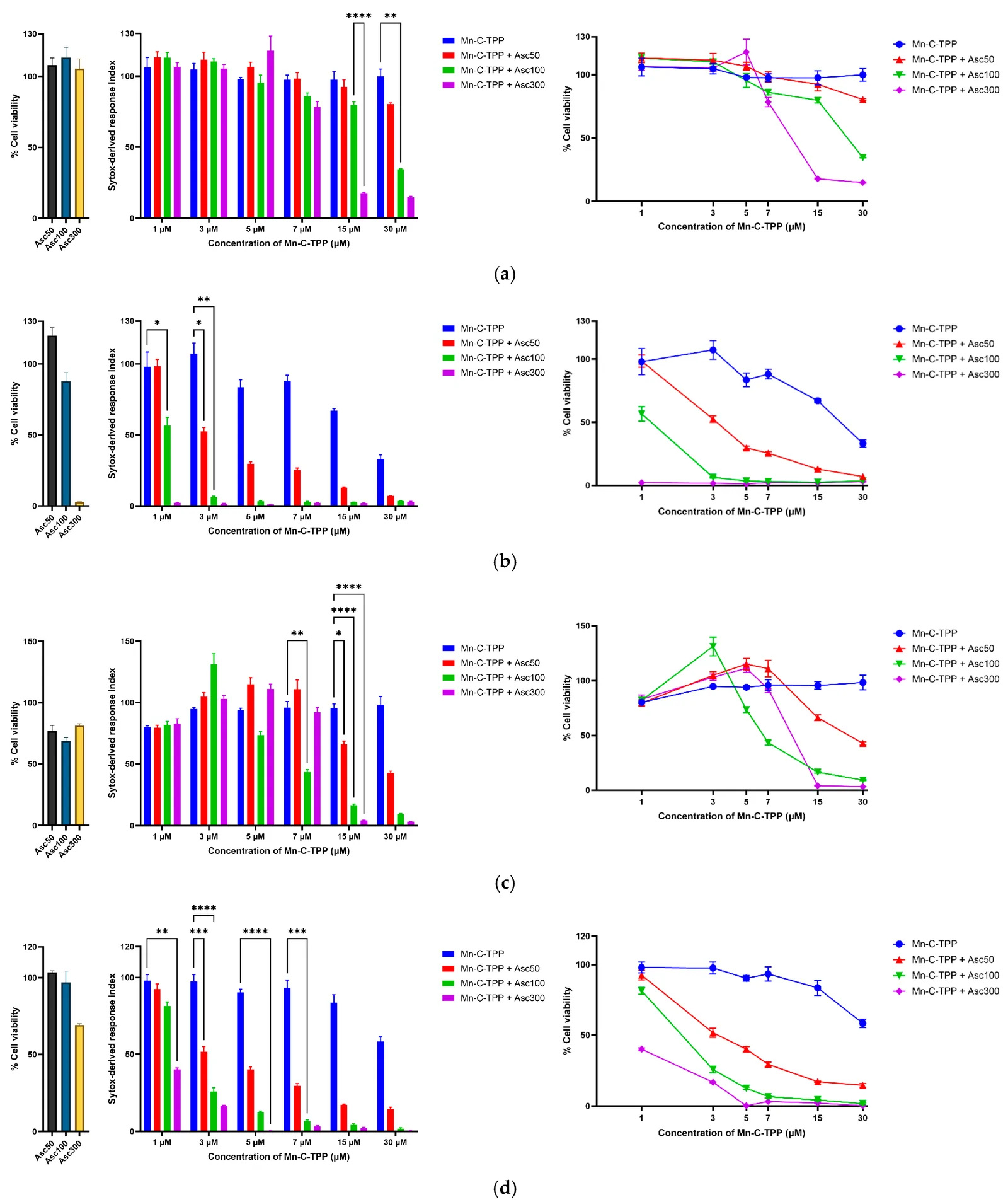
Effects of 72 h treatment with Mn-C-TPP, alone or in combination with ascorbate, on the normalized SYTOX-derived response index. (**a**) NDHF human dermal fibroblasts, (**b**) HCT116 colorectal carcinoma cells, (**c**) A549 lung adenocarcinoma cells and (**d**) HepG2 hepatoblastoma cells were treated with Mn-C-TPP (1–30 µM), alone or in combination with ascorbate (50, 100 or 300 µM). Ascorbate-only conditions are shown in separate bar plots. SYTOX Green-derived response indices were calculated using the corresponding vehicle and staurosporine controls for each cell line, assigned reference values of 100 and 0, respectively. The same Mn-C-TPP treatment data are presented as bar plots and corresponding concentration–response line plots. Data are expressed as mean ± SD (*n* = 3 independent experiments). Statistical analysis was performed using two-way repeated-measures ANOVA followed by Tukey’s multiple-comparison test. Brackets identify the groups compared. For clarity, only selected statistically significant pairwise comparisons are annotated in the main figure. The complete pairwise results, including nonsignificant comparisons, are provided in Supplementary Figures S1–S4. Asterisks indicate multiplicity-adjusted *p* values: * *p* < 0.05, ** *p* < 0.01, *** *p* < 0.001 and **** *p* < 0.0001.

After 72 h of exposure, NDHFs (Figure 1a) showed little evidence of viability loss when Mn-C-TPP (≤30 µM) or ascorbate (≤300 µM) were administered individually, with decreases in response index observed mainly under higher-dose combination conditions. Specifically, a marked decrease in the index occurred with 30 µM Mn-C-TPP + 100 µM ascorbate, while pronounced decreases were observed with 15–30 µM Mn-C-TPP + 300 µM ascorbate. At Mn-C-TPP concentrations ≤5 µM, the NDHF response index remained close to the vehicle reference across all ascorbate doses, whereas modest reductions were observed at 7 µM Mn-C-TPP with 100–300 µM ascorbate.

In HCT116 cells (Figure 1b), low concentrations of Mn-C-TPP or ascorbate alone did not appreciably affect viability as assessed by SYTOX Green. With 50 µM ascorbate, the response index remained close to the vehicle reference at 1 µM Mn-C-TPP and decreased progressively at 3–30 µM. With 100 µM ascorbate, the index was already reduced at 1 µM Mn-C-TPP and reached low values at ≥3 µM. At 300 µM ascorbate, the index was low with ascorbate alone and across all Mn-C-TPP doses.

In A549 cells (Figure 1c), Mn-C-TPP or ascorbate alone produced only limited changes across the tested ranges. With 50 µM ascorbate, 1–7 µM Mn-C-TPP did not consistently lower the index relative to Mn-C-TPP alone, whereas 15–30 µM decreased the response index; with 100 µM ascorbate, reductions emerged at 5 µM Mn-C-TPP and became more pronounced at ≥7 µM. At 5–7 µM Mn-C-TPP, the index was higher with 300 µM than with 100 µM ascorbate, but 15–30 µM Mn-C-TPP resulted in low index values. Under selected conditions in which NDHF response indices remained close to the vehicle reference, A549 cells exhibited lower response indices.

In HepG2 cells (Figure 1d), co-treatment generally produced concentration-dependent changes consistent with reduced viability. With 50 µM ascorbate, the response index remained close to the vehicle reference at 1 µM Mn-C-TPP, decreased substantially at 3 µM, and declined further at higher Mn-C-TPP doses. With 100 µM ascorbate, a reduction was already apparent at 1 µM Mn-C-TPP and became more pronounced at ≥3 µM. At 300 µM ascorbate, the index was markedly reduced even at 1 µM Mn-C-TPP and remained low at ≥3 µM. Increasing ascorbate from 50 to 300 µM generally enhanced the reduction in the response index, consistent with greater loss of viability, at matched Mn-C-TPP concentrations.

### 2.2. H_2_O_2_-Associated Luminescence

**Figure 2:**
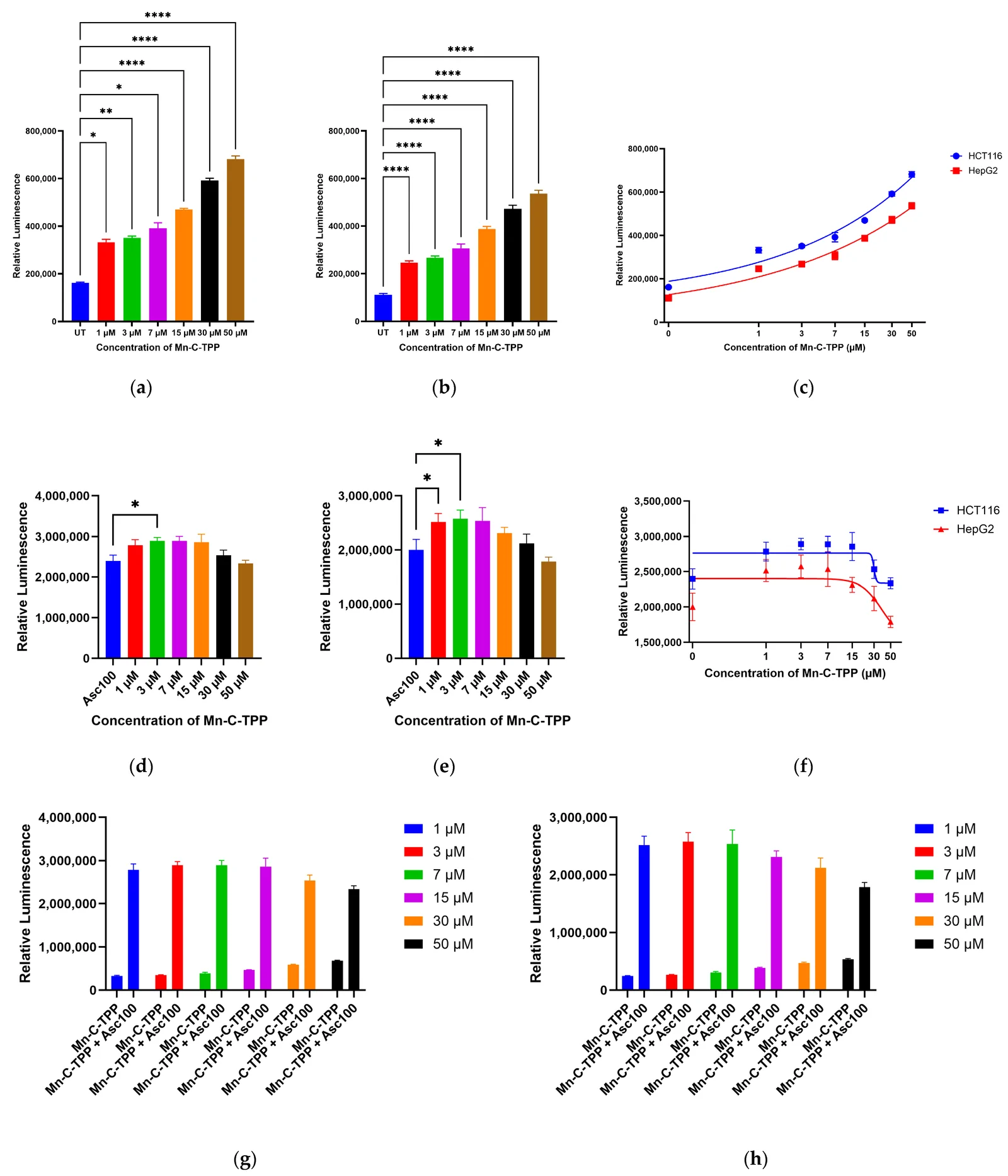
H_2_O_2_-associated luminescence in HCT116 and HepG2 cell cultures following 6 h treatment with Mn-C-TPP, alone or in combination with ascorbate. (**a**,**b**) Luminescence in HCT116 (**a**) and HepG2 (**b**) cells treated with Mn-C-TPP (1–50 µM) alone. (**c**) Corresponding concentration–response plots comparing the two cell lines. (**d**,**e**) Luminescence in HCT116 (**d**) and HepG2 (**e**) cells treated with Mn-C-TPP (1–50 µM) in combination with ascorbate (100 µM); ascorbate-only conditions are shown as the corresponding controls. (**f**) Corresponding concentration–response plots for the combination treatments. Zero Mn-C-TPP concentration represents the UT control in (**c**) and ascorbate alone in (**f**). (**g**,**h**) The same data are replotted side by side to illustrate the magnitude of the observed differences between Mn-C-TPP alone and Mn-C-TPP combined with ascorbate (100 µM) at each Mn-C-TPP concentration in HCT116 (**g**) and HepG2 (**h**) cells. H_2_O_2_-associated luminescence was measured using the ROS-Glo™ H_2_O_2_ Assay and expressed as relative luminescence units (RLU). Data are expressed as mean ± SD (*n* = 3 independent experiments). Statistical analysis was performed using one-way repeated-measures ANOVA followed by Tukey’s multiple-comparison test. Brackets identify the groups compared. An expanded version displaying all statistically significant pairwise comparisons identified by Tukey’s test is provided in Supplementary Figures S5–S8. Asterisks indicate multiplicity-adjusted *p* values: * *p* < 0.05, ** *p* < 0.01 and **** *p* < 0.0001. UT, control without Mn-C-TPP or ascorbate; Asc100, ascorbate alone (100 µM).

HCT116 and HepG2 cells were exposed for 6 h to Mn-C-TPP alone or in combination with 100 µM ascorbate, with vehicle and ascorbate-only conditions included as controls. H_2_O_2_-associated luminescence in the cultures was assessed using the ROS-Glo™ H_2_O_2_ Assay. In HCT116 cells, Mn-C-TPP across 1–50 µM led to a concentration-dependent increase in H_2_O_2_-associated luminescence, with higher mean signals than vehicle at 1 µM and the largest mean signals at 30–50 µM (Figure 2). HepG2 cells showed a similar pattern, with lower baseline luminescence and a gradual increase across the concentration range, with higher values at 30–50 µM.

In the presence of 100 µM ascorbate, H_2_O_2_-associated luminescence after 6 h increased at low Mn-C-TPP concentrations and declined at higher concentrations in both cell lines (Figure 2d–f). Relative to ascorbate alone, higher mean signals were observed at 1–30 µM Mn-C-TPP in both cell lines. Compared with ascorbate alone, the increase was statistically significant at 3 µM Mn-C-TPP in HCT116 cells (*p* = 0.0437) and at 1 and 3 µM in HepG2 cells (*p* = 0.0133 and 0.0122, respectively). At each matched Mn-C-TPP concentration, the combination produced a higher mean luminescence signal than Mn-C-TPP alone (Figure 2g,h).

### 2.3. Caspase-3/7 Activation

**Figure 3:**
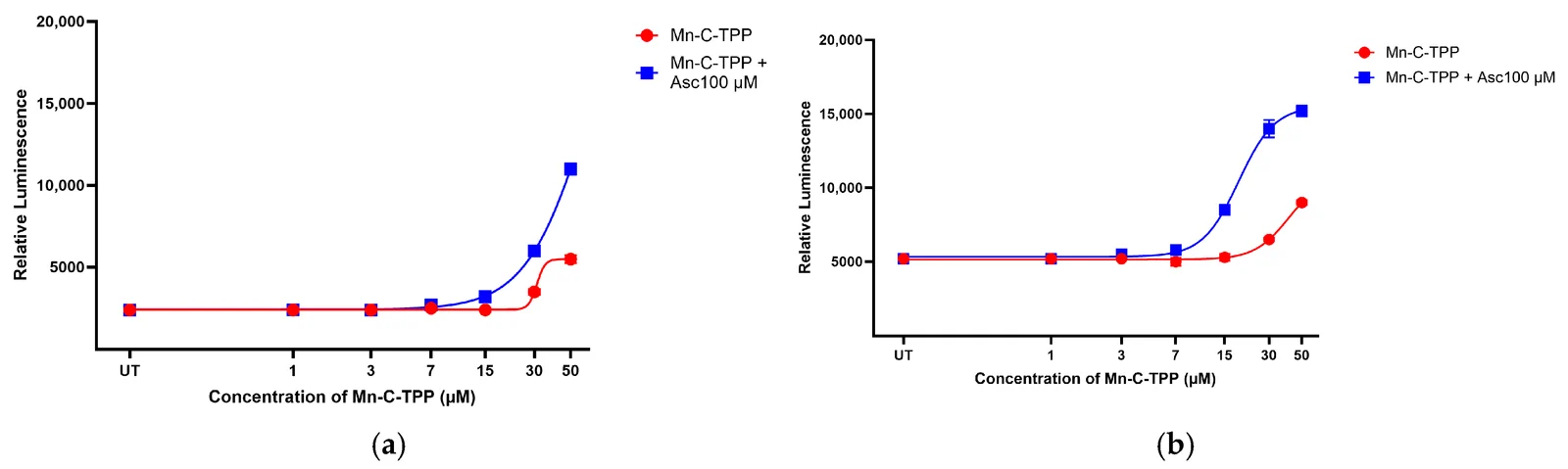
Caspase-3/7 activation in (**a**) HCT116 and (**b**) HepG2 cells after Mn-C-TPP ± ascorbate treatment. Cells were exposed for 16 h to Mn-C-TPP (1–50 µM), either alone or in combination with ascorbate (100 µM). Caspase-3/7 activity was quantified using the Caspase-Glo^®^ 3/7 luminescent assay and is expressed as relative luminescence units. In HCT116 cells, Mn-C-TPP induced a dose-dependent increase in caspase-3/7 activation, with higher mean signals in the presence of ascorbate at 15–50 µM. In HepG2 cells, a similar trend was observed, with higher baseline and treatment-associated luminescence signals. Data are expressed as mean ± SD (*n* = 3 independent experiments). Statistical analysis was performed using two-way ANOVA followed by Tukey’s multiple-comparison test. UT: untreated/vehicle control.

After 16 h of exposure to Mn-C-TPP alone or in combination with 100 µM ascorbate, caspase-3/7 activity was measured in HCT116 and HepG2 cells. In HCT116, baseline caspase activity was low. Mn-C-TPP alone led to minimal changes at 1–15 µM, with activity increasing at 30–50 µM. In the presence of ascorbate, increases in caspase activity were observed at lower Mn-C-TPP concentrations, with higher values at 15–50 µM than with Mn-C-TPP alone (Figure 3).

A similar pattern was observed in HepG2 cells, with higher baseline and treatment-associated luminescence signals than in HCT116 cells. These data indicate that Mn-C-TPP triggers caspase-3/7 activation in a concentration-dependent manner and that ascorbate is associated with higher mean signals at selected concentrations. In both cell lines, z-VAD-fmk (20 µM) reduced the treatment-associated Caspase-Glo 3/7 signal with Mn-C-TPP alone and with Mn-C-TPP plus 100 µM ascorbate, relative to the corresponding inhibitor-free conditions (Supplementary Figures S9–S12).

### 2.4. Mitochondrial Membrane Potential

**Figure 4:**
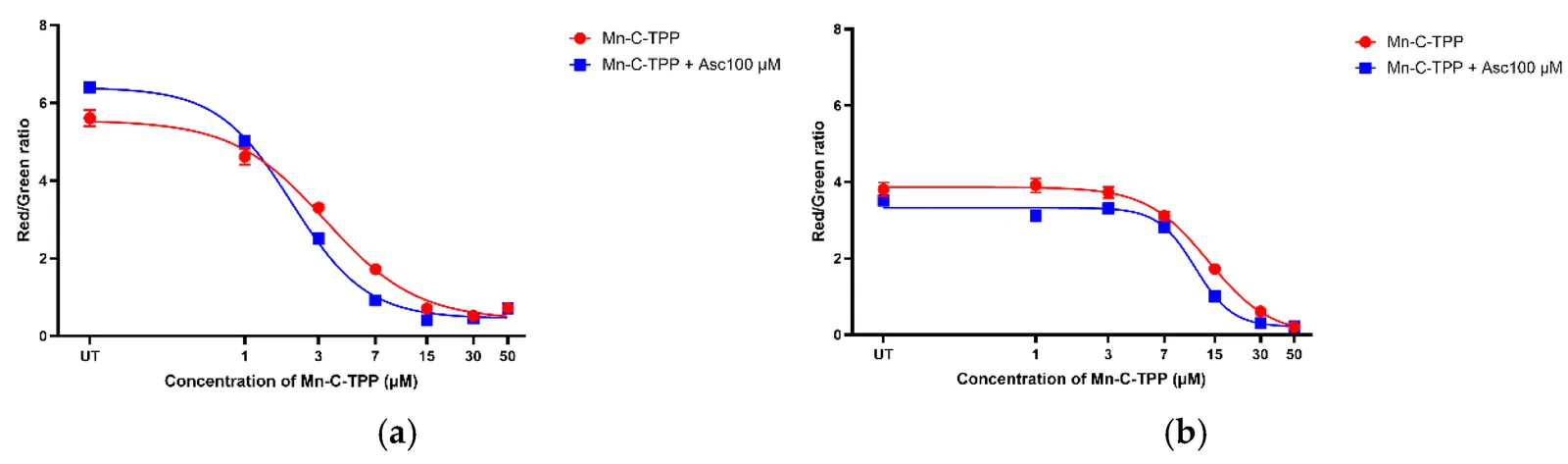
Effect of Mn-C-TPP ± ascorbate on ΔΨm in HCT116 cells. (**a**) ΔΨm changes in HCT116 cells treated for 2 h with Mn-C-TPP (1–50 µM) alone or with ascorbate (100 µM). (**b**) ΔΨm changes in HCT116 cells treated for 16 h under the same conditions. HCT116 cells were exposed to increasing Mn-C-TPP concentrations (1–50 µM), either alone or combined with ascorbate (100 µM). ΔΨm was assessed at each time point using JC-1 fluorescence, with results expressed as red-to-green fluorescence ratios. Both time points demonstrated an overall concentration-dependent decrease in the JC-1 ratio, with lower ratios in the combination group at intermediate Mn-C-TPP concentrations, consistent with mitochondrial depolarization. Data are expressed as mean ± SD (*n* = 3 independent experiments). Statistical analysis was performed using two-way ANOVA followed by Tukey’s multiple-comparison test. UT: untreated/vehicle control.

Mitochondrial membrane potential was assessed by JC-1 fluorescence in HCT116 cells exposed to Mn-C-TPP with or without 100 µM ascorbate for 2 h and 16 h. At 2 h, the JC-1 red-to-green ratio showed an overall decrease with increasing Mn-C-TPP concentration. Control values were approximately 5.5–6.5, whereas 7 µM Mn-C-TPP alone reduced the ratio to about 1.7, and 15–50 µM reduced it further to approximately 0.5–1.0. Co-treatment with ascorbate resulted in lower ratios at 2 h than Mn-C-TPP alone at 3–15 µM (Figure 4).

At 16 h, both Mn-C-TPP alone and Mn-C-TPP plus ascorbate showed concentration-dependent declines in the JC-1 ratio, with the combination condition showing lower ratios at intermediate Mn-C-TPP concentrations. At the highest concentration, both treatments converged to similar minimal ratios. These data show that ascorbate was associated with lower JC-1 ratios at intermediate Mn-C-TPP concentrations, consistent with mitochondrial depolarization under these conditions. FCCP decreased the JC-1 red-to-green fluorescence ratio relative to vehicle-treated cells, providing a positive control for mitochondrial depolarization (Supplementary Figures S13 and S14).

### 2.5. Synergy Analysis

Combination responses were examined using SynergyFinder 3.0 and the ZIP model.

**Figure 5:**
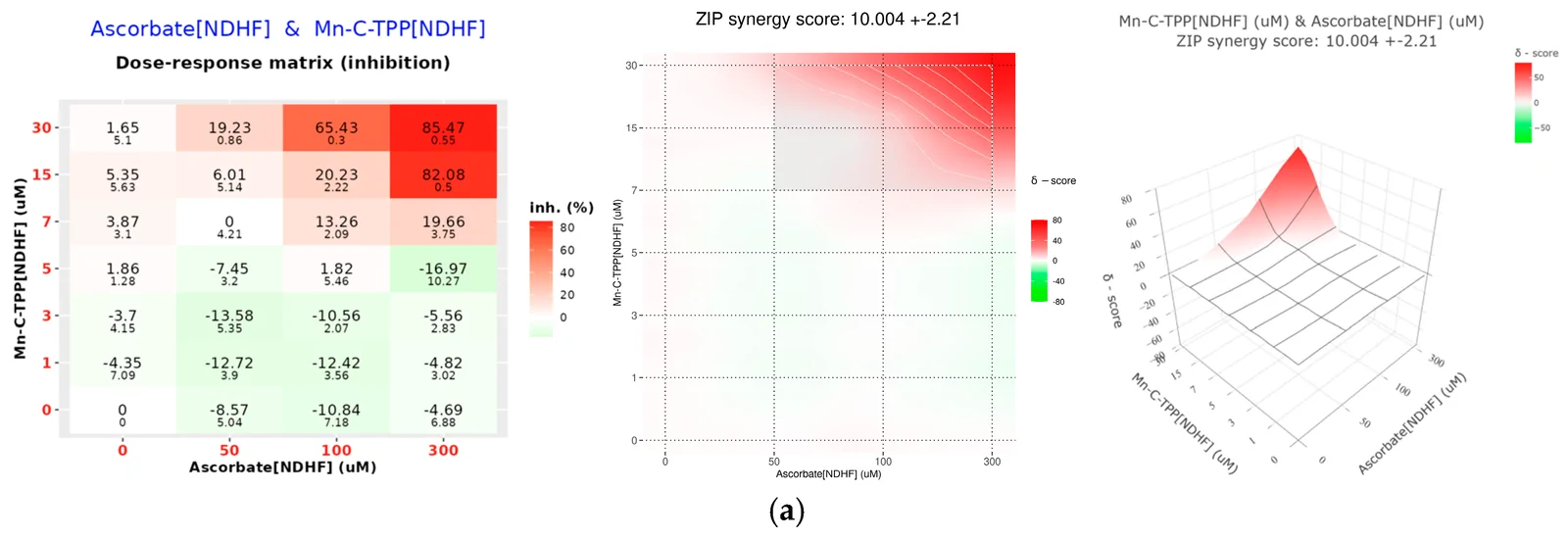

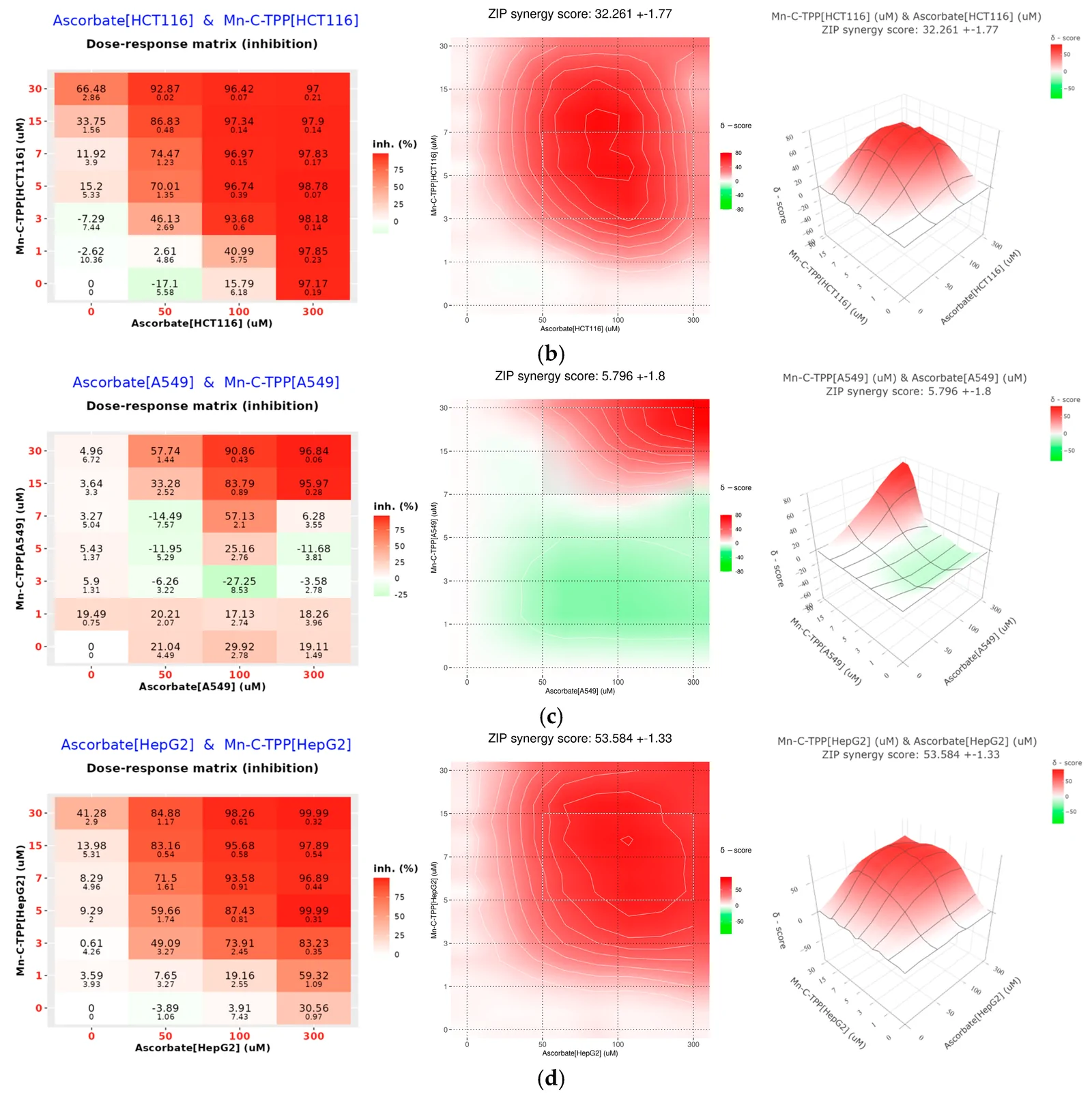
Dose–response matrices (left panels), two-dimensional (2D) synergy maps (middle panels), and three-dimensional (3D) synergy surfaces (right panels) for the Mn–C–TPP and ascorbate combinations in: (**a**) NDHF, (**b**) HCT116, (**c**) A549, and (**d**) HepG2 cells following 72 h exposure, generated with SynergyFinder 3.0 using the ZIP model. The ZIP summary synergy scores are reported with 95% confidence intervals, expressed in ± notation. Color scales represent percent inhibition in the dose–response matrices and ZIP synergy score ranges in the 2D maps and 3D surfaces. The scores were NDHF: 10.004 ± 2.21, HCT116: 32.261 ± 1.77, A549: 5.796 ± 1.8, and HepG2: 53.584 ± 1.33.

After 72 h exposure, mean ZIP scores were 10.0 ± 2.2 for NDHF, 32.3 ± 1.8 for HCT116, 5.8 ± 1.8 for A549, and 53.6 ± 1.3 for HepG2. In NDHF cells, positive synergy values were limited mainly to the highest concentrations of both agents (Figure 5).

In HCT116 and HepG2 cells, synergy extended over a broader range of Mn-C-TPP and ascorbate combinations, including intermediate doses, with inhibition frequently exceeding 90%. A549 cells showed an approximately additive overall ZIP response (mean score 5.8), despite localized positive interactions, whereas the NDHF mean score was borderline at approximately 10.

Summary synergy scores exceeded +10 in HCT116 and HepG2 under all four reference models. In A549, only the Loewe summary score exceeded +10 (12.793), whereas ZIP, Bliss and HSA scores remained within the approximately additive range; NDHF showed additive-to-borderline summary scores across the models. Results from the complementary Bliss, Loewe, and HSA analyses are provided in the Supplementary Materials Figures S15–S17.

## 3. Discussion

At pharmacological concentrations achieved only by intravenous administration, ascorbic acid can shift from its conventional antioxidant role to a pro-oxidant behavior, generating reactive oxygen species (ROS), particularly hydrogen peroxide (H_2_O_2_), in the tumor microenvironment [43,44]. However, the cytotoxic effects of ascorbate depend on its concentration, the cellular model, and the experimental conditions [41,45]. To increase efficacy and selectivity, several groups have developed exogenous catalysts—most notably manganese porphyrins—that can accumulate preferentially in tumors and act as SOD-mimetic and peroxidase-like catalysts, thereby accelerating ascorbate oxidation and H_2_O_2_ production. In these Mn-porphyrin/ascorbate systems, ascorbate can act as a prodrug for oxidative stress, with preferential cytotoxicity toward malignant cells reported under specific experimental conditions [40,44,46].

Building on this concept, and on the growing interest in mitochondria-targeted redox-active compounds [47,48], we synthesized a manganese–curcumin–triphenylphosphonium complex (Mn-C-TPP), in which manganese is coordinated by a curcumin ligand and linked to a lipophilic triphenylphosphonium (TPP^+^) cation [33]. This architecture combines (1) a manganese center, with SOD-mimetic activity reported for the complex in previous work [33]; (2) curcumin as an antitumor pharmacophore that modulates signaling, inflammation, and redox balance; and (3) a TPP^+^ moiety intended to promote mitochondrial accumulation by exploiting the negative mitochondrial membrane potential (ΔΨm) [31,49]. This membrane potential provides a basis for the electrophoretic accumulation of lipophilic TPP^+^-conjugated species within mitochondria [31,50]. Our approach is consistent with the Mn-porphyrin/ascorbate paradigm, in which Mn redox centers can catalyze ascorbate oxidation and amplify H_2_O_2_-driven oxidative stress, resulting in synergistic cancer cell killing [41]. A key feature of the present system is the mitochondrial targeting intended through TPP^+^ conjugation, which is expected to increase local compound concentration within the mitochondrial compartment. Within this framework, Mn-C-TPP alone reduced the SYTOX-derived response index in HCT116 and HepG2 cells, whereas A549 cells and normal dermal fibroblasts were less affected. Ascorbate sensitized tumor cells to Mn-C-TPP at selected combinations, including concentrations with modest single-agent activity. The observed H_2_O_2_-associated luminescence, mitochondrial depolarization, and caspase-3/7 activation support further investigation of a redox-related mechanism for Mn-C-TPP–ascorbate cytotoxicity.

The included lines were selected on the basis of previous studies of pharmacological ascorbate and oxidative stress mechanisms [36]: (i) HCT116 colorectal cancer, which is a KRAS-mutant model used in foundational work demonstrating susceptibility of KRAS/BRAF-mutant colorectal cancer cells to high-dose vitamin C through redox disruption and oxidative stress mechanisms [51]. HCT116 is a well-characterized colorectal cancer cell line widely used in studies of curcumin and curcumin-derived compounds, providing a robust contextual framework for interpreting responses to Mn-C-TPP. Previous reports describe cytotoxic and pro-apoptotic effects of curcumin in HCT116 cells [52,53]. On this basis, HCT116 provided a biologically relevant and technically reproducible reference for interpreting the combined-treatment responses; (ii) A549 non–small cell lung cancer, a canonical NSCLC model in which pharmacological ascorbate-induced cytotoxicity has been linked to extracellular H_2_O_2_, as supported by catalase protection [54]; and (iii) HepG2 hepatoblastoma, a widely used liver tumor model in which pharmacological ascorbate has been investigated for effects on oxidative responses and mitochondrial membrane potential [55] and which exhibits distinctive antioxidant/redox enzyme activity profiles relevant to redox buffering [56].

Results from our SYTOX Green viability assay indicate a pattern consistent with synergistic interaction between Mn-C-TPP and ascorbate at selected combinations. At low-micromolar concentrations, Mn-C-TPP alone produced little cytotoxicity, and ascorbate alone (≤100 μM, 72 h) caused only modest viability loss in most cancer cell lines. In contrast, combined treatment produced a marked and statistically significant decrease in the SYTOX-derived response index, consistent with reduced viability, particularly in HCT116 colorectal carcinoma cells. Thus, selected combinations reduced the response index at concentrations at which the individual agents had limited activity.

Our data further suggest that cytotoxic effects can be obtained with submillimolar ascorbate combined with Mn-C-TPP. Previous Mn-porphyrin/ascorbate studies have used various ascorbate concentrations, including approximately 0.5–3.3 mM under different treatment schedules [40,41,46]. By contrast, in our Mn-C-TPP experiments, marked reductions in the SYTOX-derived response index were observed with 100 µM ascorbate plus low-micromolar Mn-C-TPP in HCT116 and HepG2 cells, assessed after 72 h. Differences in exposure duration, catalyst structure, cell model, cell density, culture conditions, and assay endpoints must be considered when comparing apparent ascorbate requirements [55]. In addition, millimolar ascorbate can acidify the culture medium and induce non-specific stress [57], whereas 0.1 mM (100 μM) lies within the range considered attainable in tumor interstitial fluid during intravenous ascorbate therapy [58]. Within this more prolonged, lower-dose framework, Mn-C-TPP–ascorbate combinations showed activity at submillimolar ascorbate concentrations under the conditions tested.

The differential sensitivity of malignant and non-malignant cells is central to the translational potential of Mn-C-TPP plus ascorbate [46,59,60]. In normal human dermal fibroblasts exposed to conditions that were clearly cytotoxic for several tumor cell lines, viability was largely preserved as assessed by SYTOX Green: 3 μM Mn-C-TPP plus 100 μM ascorbate, which substantially reduced the response index in HCT116 and HepG2 cells, maintained the fibroblast response index close to the vehicle reference (100). Reductions in the fibroblast response index were observed at higher combination doses, with pronounced decreases at 30 µM Mn-C-TPP plus 100 µM ascorbate and at 15–30 µM Mn-C-TPP plus 300 µM ascorbate. Thus, these findings demonstrate concentration-dependent differences in sensitivity between the tested cancer cell lines and NDHFs, but they do not establish general selectivity for malignant cells. Published Mn-porphyrin/ascorbate studies have reported preferential effects in malignant cells under specific experimental conditions [40,41]; nevertheless, confirmation of tumor selectivity for Mn-C-TPP plus ascorbate will require comparisons with tissue-matched non-malignant epithelial, pulmonary, and hepatocyte-like models. Moreover, differences in redox buffering or compound uptake may contribute to the observed responses [8,61,62], but these determinants were not directly evaluated in the present models.

Our findings also highlight cell-line-dependent variability in response. HCT116 colorectal carcinoma cells were highly responsive to the combination. High-dose vitamin C has been reported to preferentially kill KRAS/BRAF-mutant colorectal cancer cells through redox disruption and inhibition of GAPDH [51]. The stronger overall interaction in HCT116 and HepG2 than in A549 may reflect differences in H_2_O_2_ clearance, antioxidant buffering, and metabolic state [36,51]. These factors could modulate the peroxide-dependent injury described in Mn-porphyrin/ascorbate studies [40,41], although their contribution to the present response patterns was not directly evaluated. This heterogeneity is consistent with variable responses of NSCLC models to redox-active manganese compounds [63] and with the enhanced cytotoxicity reported for rucosopasem combined with ascorbate in A549 cells [64]. These differences support the view that cellular redox biology and antioxidant capacity may modulate the efficacy of Mn-C-TPP–ascorbate combinations. Further studies will be needed to identify determinants of response and to evaluate combinations with agents that weaken antioxidant defenses in less responsive models [38,60,65].

The cytotoxicity findings were complemented by measurements of oxidative and mitochondrial responses in selected models. In HCT116 and HepG2 cells, we observed a concentration-dependent increase in H_2_O_2_-associated luminescence after 6 h of Mn-C-TPP alone, using the ROS-Glo™ H_2_O_2_ Assay. The four-condition comparison also showed a substantial signal with ascorbate alone and higher mean luminescence with selected low-micromolar Mn-C-TPP combinations. The additional increase relative to ascorbate alone was not maintained uniformly across the concentration range. The comparison with ascorbate alone identifies selected low Mn-C-TPP concentrations at which the combination increases H_2_O_2_-associated luminescence beyond the ascorbate-only response.

Caspase-3/7 activity was assessed after 16 h in both cell lines, with increases more apparent at 30–50 µM Mn-C-TPP alone and at 15–50 µM in the presence of 100 µM ascorbate. Mitochondrial membrane potential was assessed only in HCT116 cells, where the JC-1 red-to-green ratio decreased at both 2 h and 16 h, with lower ratios in the combination group at intermediate Mn-C-TPP concentrations. These observations are consistent with oxidative and mitochondrial perturbations accompanied by caspase activation. Previous Mn-porphyrin/ascorbate studies have also implicated caspase-independent mechanisms, including AIF-associated cell death [40]. Recent preclinical studies connect mitochondrial perturbation with diverse cell-death pathways. TPP^+^-modified fatty-acid nanoassemblies induced apoptosis and paraptosis [66], while photoactivated Z1 nanoparticles induced apoptosis and pyroptosis [67]. The natural compound kuwanon H impaired autophagic flux [68], whereas the ceramide analog LCL768 promoted lethal mitophagy [69]. These findings suggest mitochondrial quality control and cell-death signaling as additional potential determinants of treatment sensitivity. Their contribution to the heterogeneous responses observed with Mn-C-TPP plus ascorbate requires direct investigation before translational conclusions can be drawn. Together, the present findings support further investigation of a mitochondrial redox mechanism for Mn-C-TPP–ascorbate cytotoxicity; they do not establish the causal order of the measured responses or demonstrate that cell death is primarily caspase-dependent.

In line with the SYTOX Green viability assay results, formal combination analysis with SynergyFinder 3.0 (ZIP model) suggested pronounced positive interactions between Mn-C-TPP and ascorbate in HCT116 and HepG2 cells at the level of the SYTOX-derived response index. After 72 h exposure, mean ZIP scores were approximately 32 in HCT116 and 54 in HepG2. A549 cells showed a low overall score (approximately 6), within the commonly used additive range, despite localized positive and negative interactions, whereas NDHFs had a borderline mean score (approximately 10), with the more pronounced positive interactions mainly at high-dose combinations. These quantitative patterns indicate that the interaction between Mn-C-TPP and ascorbate differed among the tested cell lines, being strongest in HCT116 and HepG2, weaker overall in A549, and restricted mainly to higher-dose combinations in NDHFs. Agreement across ZIP, Bliss, Loewe and HSA strengthens the interpretation of pronounced positive interactions in HCT116 and HepG2 within the same response dataset. The model-dependent classification in A549 further highlights the value of examining complementary reference models alongside the concentration-specific response pattern. Published Mn-porphyrin/ascorbate studies have reported preferential cytotoxicity in malignant cells under specific experimental conditions [40,41,46], but whether Mn-C-TPP exhibits a comparable pattern requires validation in additional, tissue-relevant non-malignant models.

The present study has certain limitations. Our research was performed on 2D in vitro models, which lack the architectural and metabolic complexity of tumor microenvironments; therefore, future work will evaluate Mn-C-TPP plus ascorbate in 3D spheroids, patient-derived organoids, and in vivo xenograft models to better define efficacy, drug penetration, and the therapeutic window. Selectivity was assessed using NDHF cells as the non-malignant comparator, and future studies will incorporate additional primary normal cells, including epithelial and hepatocyte-like models, to strengthen translational conclusions. Mechanistic assays focused on early functional endpoints—culture H_2_O_2_-associated luminescence, mitochondrial membrane potential (ΔΨm), and caspase-3/7 activation—in selected cancer cell lines. ROS-Glo does not resolve the cellular or subcellular origin of H_2_O_2_. Although the four-condition comparison showed higher H_2_O_2_-associated luminescence with selected combinations than with ascorbate alone, these measurements do not establish whether H_2_O_2_ mediates the observed cytotoxicity. Direct mitochondrial accumulation and catalytic activity of Mn-C-TPP were not measured in the present experiments. In future studies, we will also integrate compartment-resolved mitochondrial redox measurements, including mitochondria-targeted ROS probes (e.g., MitoSOX), genetically encoded redox sensors (mito-HyPer/roGFP), and biochemical redox couples (GSH/GSSG and NAD(P)H/NAD(P)^+^), to achieve higher mechanistic resolution. Moreover, compartment-specific H_2_O_2_ flux and ascorbate decay kinetics were not directly measured; future experiments will quantify these using complementary H_2_O_2_ assays and HPLC-based ascorbate time-course analyses. The SYTOX-derived response index was normalized using vehicle and staurosporine controls and does not represent a direct cell count or an independently established absolute proportion of viable cells. Orthogonal validation using clonogenic survival assays and independent apoptosis/necrosis markers would further substantiate the interaction framework.

## 4. Materials and Methods

### 4.1. Chemicals and Reagents

Curcumin, manganese(II) acetate, (4-bromobutyl)triphenylphosphonium bromide, sodium hydride (60% dispersion in mineral oil), anhydrous *N,N*-dimethylformamide (DMF), dimethyl sulfoxide (DMSO), and absolute ethanol were purchased from Sigma-Aldrich/Merck (Darmstadt, Germany) and used as received for the synthesis of Mn-C-TPP. Sodium L-ascorbate (Sigma-Aldrich/Merck) was prepared immediately prior to each experiment by dissolving the powder in sterile water to obtain a concentrated stock solution, which was then diluted into complete growth medium to the indicated final concentrations. Ascorbate solutions were prepared immediately before addition to cells and used without prolonged storage.

SYTOX™ Green nucleic acid stain, 5,5′,6,6′-tetrachloro-1,1′,3,3′-tetraethylbenzimidazolylcarbocyanine iodide (JC-1), phenol-red-free Hanks’ Balanced Salt Solution (HBSS), cell-culture media including McCoy’s 5A medium, Dulbecco’s Modified Eagle Medium (DMEM), and Ham’s F-12/F-12K nutrient mixtures, as well as fetal bovine serum (FBS), were obtained from Thermo Fisher Scientific (Waltham, MA, USA).

The ROS-Glo™ H_2_O_2_ Assay kit and the Caspase-Glo^®^ 3/7 luminescent assay kit were obtained from Promega (Madison, WI, USA). The pan-caspase inhibitor benzyloxycarbonyl-Val-Ala-Asp-fluoromethyl ketone (z-VAD-fmk), the apoptosis inducer staurosporine, and the mitochondrial uncoupler carbonyl cyanide 4-(trifluoromethoxy)phenylhydrazone (FCCP) were purchased from Sigma-Aldrich/Merck.

Black, clear-bottom 96-well microplates for fluorescence measurements and white, opaque 96-well microplates for luminescence measurements were obtained from Corning/Costar (Corning, NY, USA). Unless otherwise specified, all reagents were of analytical grade.

### 4.2. Chemical Synthesis of Mn-C-TPP

Mn-C-TPP was synthesized and purified as described in our previous study [33]. The present biological experiments and the previously reported analytical characterization were performed using Mn-C-TPP from a single synthesis batch. Briefly, the synthesis involved a two-step reaction. First, curcumin (C) was complexed with manganese(II) acetate in ethanol under reflux conditions to yield the Mn-C intermediate [33]. In the second step, the Mn-C complex was alkylated with (4-bromobutyl)triphenylphosphonium bromide in the presence of sodium hydride using dry *N,N*-dimethylformamide (DMF) as the solvent, under an inert atmosphere. The resulting compound, Mn-C-TPP, was isolated by filtration and washed with cold ethanol. Stock solutions of Mn-C-TPP were prepared in dimethyl sulfoxide (DMSO) at a concentration of 50 mM and stored at −80 °C in an ultra-low temperature freezer (Forma 900 Series, Thermo Fisher Scientific, Waltham, MA, USA) and thawed immediately before use. Working concentrations of Mn-C-TPP were obtained by dilution of the stock into pre-warmed complete growth medium; the final DMSO concentration was maintained at ≤0.1% (*v*/*v*) in all treatment conditions, and matched vehicle controls were included. As reported earlier, the compound is stable for at least one month in solid form, whereas solutions exhibit reduced biological activity after 24 h at room temperature.

### 4.3. Cell Lines

All cell lines were obtained from Cell Lines Service (CLS, Eppelheim, Germany). HCT116 (human colorectal carcinoma) cells were maintained in McCoy’s 5A medium, A549 (human lung carcinoma) in a 1:1 mixture of DMEM and Ham’s F-12 nutrient medium, HepG2 (human hepatoblastoma) in Ham’s F-12 medium, and normal dermal human fibroblasts (NDHFs) in DMEM. All media were supplemented with 10% fetal bovine serum (FBS), and cells were incubated at 37 °C in a humidified atmosphere of 5% CO_2_ in a CO_2_ incubator (Thermo Scientific HERAcell 150i, Thermo Fisher Scientific, Waltham, MA, USA). Cell-culture media (McCoy’s 5A, DMEM, Ham’s F-12) and FBS were from Thermo Fisher Scientific (Gibco). All supplemented media are referred to as growth media. HCT116, A549, and HepG2 cells were authenticated by supplier-performed STR profiling and used within 5–20 passages after thawing. NDHF provenance was documented by supplier records, and fibroblasts were used at passages 3–8. No additional in-house authentication was performed. All cultures were tested for mycoplasma using a PCR-based assay upon receipt and before each experimental series, with negative results. 

### 4.4. Treatment of Cells for Determination of Viability

The effects of Mn-C-TPP and ascorbate were evaluated in three malignant human cell lines: HCT116, A549 and HepG2. On day 0, 96-well plates were seeded with 10^3^ cells/well and allowed to adhere for 24 h in growth medium according to the suppliers’ instructions. Cells were subsequently treated for 72 h with Mn-C-TPP or freshly prepared ascorbate in growth media, either individually or in combination at the indicated concentrations, with ≤0.1% (*v*/*v*) DMSO as vehicle. Treatment duration and ascorbate concentrations were selected to capture cumulative cytotoxicity and to define the interaction window. The dose–response matrices presented in Figure 5 used Mn-C-TPP at 1, 3, 5, 7, 15, or 30 µM combined with ascorbate at 50, 100, or 300 µM, together with the corresponding single-agent and vehicle controls. Based on the preliminary SYTOX Green viability assay results and the biological relevance of the selected concentration [70], 100 µM ascorbate was chosen as a common working condition for subsequent mechanistic assays. A 72 h exposure window was selected to assess cumulative cytotoxicity while aiming to limit over-confluence of vehicle-treated controls. NDHFs were included as a non-malignant comparator to provide an initial assessment of differential cellular sensitivity under the tested conditions.

Cell viability after 72 h of treatment was assessed using the SYTOX™ Green nucleic acid stain (Thermo Fisher Scientific), which selectively labels non-viable cells with compromised plasma membranes. Following treatment, cells were washed with phosphate-buffered saline (PBS) and incubated with SYTOX™ Green working solution at a final concentration of 1 µM for 15 min at room temperature, protected from light. Detached cells were recovered and combined with the corresponding adherent-cell fraction before fluorescence measurement. All fluorescence and luminescence measurements were acquired using a multimode microplate reader (Infinite 200 PRO, Tecan Group Ltd., Männedorf, Switzerland). SYTOX™ Green fluorescence was recorded at excitation and emission wavelengths of 504 and 523 nm, respectively. Background fluorescence from dye-only controls was subtracted. To assess potential chemical interference, wells containing Mn-C-TPP alone (without cells) and vehicle controls were processed in parallel. For all assays, a no-drug control (0 µM Mn-C-TPP and 0 µM ascorbate) was included on each plate. Because Mn-C-TPP stocks were prepared in DMSO and ascorbate stocks in sterile water, no-drug control wells received equivalent volumes of vehicle (final DMSO ≤0.1% *v*/*v* and matching water volume) and served as the baseline (“untreated/vehicle control”) for normalization. Unless stated otherwise, outcomes are reported relative to this control. Staurosporine (1 µM) was included on each plate as a positive control for cell death and assay performance and as a normalization reference for the SYTOX™ Green assay. Background-corrected SYTOX™ Green fluorescence was linearly normalized using the corresponding vehicle and staurosporine controls for each cell line. Results were expressed as a SYTOX-derived viability index, with the vehicle control assigned a value of 100 and the staurosporine control a value of 0. These values define an inverse normalized fluorescence scale and do not establish the absolute proportions of viable cells in the reference or treatment conditions. Values above 100 correspond to fluorescence signals below the vehicle-control reference. All conditions were run in triplicate.

### 4.5. Evaluation of H_2_O_2_-Associated Luminescence Following 6 h Mn-C-TPP Exposure

On day 0, HCT116 and HepG2 cells were seeded into 96-well plates at 10,000 cells/well and allowed to adhere for 24 h. Cells were then exposed for 6 h to Mn-C-TPP at final concentrations of 1, 3, 7, 15, 30, or 50 µM, either alone or in combination with 100 µM sodium L-ascorbate, in complete medium containing DMSO ≤0.1% (*v*/*v*). Vehicle-treated cells and cells exposed to 100 µM sodium L-ascorbate alone were included. H_2_O_2_ levels in the cell cultures were assessed using the ROS-Glo™ H_2_O_2_ Assay (Promega, Madison, WI, USA), following the manufacturer’s homogeneous assay protocol. At the start of treatment, 20 µL of H_2_O_2_ Substrate Solution (125 µM) was added to 80 µL of cell culture and treatment medium, yielding a final substrate concentration of 25 µM in a total volume of 100 µL per well. The stated Mn-C-TPP and ascorbate concentrations refer to this final incubation volume. Cells were incubated for 6 h at 37 °C in a humidified atmosphere containing 5% CO_2_. Subsequently, 100 µL of freshly prepared ROS-Glo™ Detection Solution was added directly to each well, and the plate was incubated for 20 min at room temperature (22–25 °C). Endpoint luminescence was recorded using the multimode microplate reader described above (Infinite 200 PRO, Tecan Group Ltd., Männedorf, Switzerland). Results were expressed as relative luminescence units (RLU), with vehicle-treated cells providing the basal reference for each cell line. Data are presented as mean ± SD (*n* = 3).

### 4.6. Caspase-3/7 Activity After 16 h Exposure to Mn-C-TPP with or Without Ascorbate

On day 0, HCT116 and HepG2 cells were seeded into white, opaque 96-well plates at 10,000 cells/well and allowed to adhere for 24 h. Cells were then treated for 16 h with Mn-C-TPP at the concentrations shown in Figure 3 (1, 3, 7, 15, 30, or 50 µM), with or without 100 µM sodium L-ascorbate (vehicle DMSO ≤0.1% *v*/*v* in all wells). Caspase-3/7 activity was quantified using a homogeneous luminescent assay (Caspase-Glo^®^ 3/7) following the manufacturer’s instructions, and luminescence was recorded using the microplate reader described above, with an integration time of 0.5–1 s per well. Luminescence was corrected for background and compound-associated signal using cell-free controls containing reagent and medium, with or without Mn-C-TPP, as appropriate. z-VAD-fmk (20 µM) was used in parallel as a caspase-inhibition control, while staurosporine (1 µM, 4 h) served as a positive control for apoptosis. Caspase-3/7 activity was expressed as RLU, with vehicle-treated cells providing the basal activity reference for each cell line. All conditions were run in triplicate across at least three independent experiments.

### 4.7. Mitochondrial Membrane Depolarization After 2 h and 16 h of Mn-C-TPP + Ascorbate Exposure

On day 0, HCT116 cells were seeded into black, clear-bottom 96-well plates at 10,000 cells/well and allowed to adhere for 24 h. Cells were then treated with Mn-C-TPP at the concentrations shown in Figure 4 (1, 3, 7, 15, 30, or 50 µM), with or without 100 µM sodium L-ascorbate (vehicle DMSO ≤0.1% *v*/*v*), for either 2 h or 16 h. At each endpoint, cultures were gently rinsed with prewarmed phenol-red-free HBSS and stained with JC-1 (10 µg/mL in HBSS; 10 min, 37 °C, protected from light); the dye was maintained during reading to preserve steady-state distribution. Fluorescence was recorded using the microplate reader described above at excitation/emission wavelengths of 485/535 nm for the green channel and 535/590 nm for the red channel. Background fluorescence from no-dye controls and compound autofluorescence, assessed using cell-free wells containing matched concentrations of Mn-C-TPP with or without ascorbate, was corrected using the corresponding controls. FCCP (10 µM, 10–15 min) was included as a depolarization control. Results were expressed as JC-1 red-to-green fluorescence ratios, with lower ratios indicating mitochondrial membrane depolarization. All conditions were assayed in triplicate in at least three independent experiments.

### 4.8. Statistical Analysis

All statistical analyses were performed using GraphPad Prism software (version 10.6; GraphPad Software, Boston, MA, USA). Data are presented as mean ± SD from at least three independent experiments, each performed in technical triplicate unless otherwise specified. For Figure 1 and Figure 2, group differences were evaluated using repeated-measures ANOVA, followed by Tukey’s post hoc multiple-comparison test. Values of *p* < 0.05 were considered statistically significant.

All statistical analyses were performed using GraphPad Prism software (version 10.6; GraphPad Software, Boston, MA, USA). Data are presented as mean ± SD from three independent experiments, each performed in technical triplicate unless otherwise specified. Technical triplicates were averaged to obtain one value per condition for each independent experiment. Normality was assessed using the Shapiro–Wilk and Kolmogorov–Smirnov tests; neither test detected statistically significant departures from normality in the datasets assessed. Treatment effects were evaluated using one-way or two-way repeated-measures analysis of variance (ANOVA), as appropriate, with the independent experiment as the matching factor, followed by Tukey’s multiple-comparison test. For two-way ANOVA, comparisons were conducted within rows and within columns, with each row and each column treated as a separate family of comparisons. Multiplicity-adjusted *p* values < 0.05 were considered statistically significant. Drug combination effects were evaluated using SynergyFinder 3.0 (Institute for Molecular Medicine Finland, University of Helsinki, Helsinki, Finland), applying the zero-interaction potency (ZIP), Bliss independence, Loewe additivity and highest single agent (HSA) reference models to the dose–response matrices. Summary synergy scores above +10 were interpreted as suggesting synergy, scores from −10 to +10 as approximately additive, and scores below −10 as suggesting antagonism. The ZIP analysis is presented in Figure 5 of the main manuscript, and the complementary Bliss, Loewe and HSA analyses are presented in Supplementary Materials Figures S15–S17.

## 5. Conclusions

This study shows that Mn-C-TPP, designed for mitochondrial targeting, produces marked reductions in the control-normalized viability index derived from SYTOX Green fluorescence when combined with ascorbate, including at selected concentrations with limited single-agent activity. Lower-dose combinations elicited greater responses in HCT116 and HepG2 cells than in NDHFs. Exploratory analyses using four reference models consistently supported pronounced positive interactions in HCT116 and HepG2, while revealing a model-dependent overall classification in A549 and additive-to-borderline responses in NDHF. Complementary assays showed increased culture H_2_O_2_-associated luminescence following Mn-C-TPP alone and higher mean signals with selected combinations than with ascorbate alone, alongside mitochondrial depolarization and caspase-3/7 activation in selected cancer-cell models. These findings provide an experimental foundation for investigating Mn-C-TPP–ascorbate and its proposed redox-related mechanism. Building on this foundation, independent survival assays, direct localization measurements and functional rescue experiments can establish how the observed cellular responses contribute to treatment effects. Evaluation in tissue-matched non-malignant models, three-dimensional systems and appropriate in vivo models can then assess whether the observed differences in sensitivity are reproducible and therapeutically relevant.

## Data Availability

The raw data supporting the conclusions of this article will be made available by the authors on request.

## Supplementary Materials

The following supporting information can be downloaded at: https://www.mdpi.com/article/10.3390/ph19101549/s1, Figure S1: Significant pairwise comparisons in NDHF cells (Figure 1a); Figure S2: Significant pairwise comparisons in HCT116 cells (Figure 1b); Figure S3: Significant pairwise comparisons in A549 cells (Figure 1c); Figure S4: Significant pairwise comparisons in HepG2 cells (Figure 1d); Figure S5: H_2_O_2_-associated luminescence comparisons in HCT116 cells treated with Mn-C-TPP alone; Figure S6: H_2_O_2_-associated luminescence comparisons in HepG2 cells treated with Mn-C-TPP alone; Figure S7: H_2_O_2_-associated luminescence comparisons in HCT116 cells treated with Mn-C-TPP plus ascorbate; Figure S8: H_2_O_2_-associated luminescence comparisons in HepG2 cells treated with Mn-C-TPP plus ascorbate; Figure S9: z-VAD-fmk controls for caspase-3/7 activity in HCT116 cells; Figure S10: z-VAD-fmk controls for caspase-3/7 activity in HepG2 cells; Figure S11: Staurosporine and z-VAD-fmk controls for the HCT116 caspase-3/7 assay; Figure S12: Staurosporine and z-VAD-fmk controls for the HepG2 caspase-3/7 assay; Figure S13: FCCP controls for the JC-1 assay in HCT116 cells at the 2 h endpoint; Figure S14: FCCP controls for the JC-1 assay in HCT116 cells at the 16 h endpoint; Figure S15: Mn-C-TPP–ascorbate interaction analysis using the Bliss model; Figure S16: Mn-C-TPP–ascorbate interaction analysis using the Loewe model; Figure S17: Mn-C-TPP–ascorbate interaction analysis using the HSA model.

## Abbreviations

The following abbreviations are used in this manuscript:

AIF	Apoptosis-inducing factor
ANOVA	Analysis of variance
Asc	Ascorbate
ATP	Adenosine triphosphate
BRAF	v-Raf murine sarcoma viral oncogene homolog B1
Mn-C-TPP	Manganese–curcumin–triphenylphosphonium complex
DMSO	Dimethyl sulfoxide
DMEM	Dulbecco’s Modified Eagle Medium
EMEM	Eagle’s Minimum Essential Medium
FCCP	Carbonyl cyanide 4-(trifluoromethoxy)phenylhydrazone
FBS	Fetal bovine serum
HBSS	Hanks’ Balanced Salt Solution
H_2_O_2_	Hydrogen peroxide
JC-1	5,5′,6,6′-Tetrachloro-1,1′,3,3′-tetraethylbenzimidazolylcarbocyanine iodide
KRAS	Kirsten rat sarcoma viral oncogene homolog
Mn-C	Manganese–curcumin intermediate complex
MMP	Mitochondrial membrane potential
NDHF	Normal dermal human fibroblasts
NRF2	Nuclear factor erythroid 2–related factor 2
PBS	Phosphate-buffered saline
RLU	Relative luminescence units
ROS	Reactive oxygen species
SOD	Superoxide dismutase
SYTOX Green	Cell-impermeant nucleic acid stain for dead-cell quantification
TPP^+^	Triphenylphosphonium cation
z-VAD-fmk	Benzyloxycarbonyl-Val-Ala-Asp-fluoromethyl ketone (pan-caspase inhibitor)
ZIP	Zero Interaction Potency (synergy model)
