# Supplementary material for: Ascorbate Enhances the Cytotoxicity of the Manganese–Curcumin–Triphenylphosphonium Complex in Human Cancer Cells
Source: Pharmaceuticals (Basel). 2026 Sep 30;19(10):1549. doi: 10.3390/ph19101549 (PMC13708449; doi:10.3390/ph19101549)
Supplement: Supplementary file 1 [file pharmaceuticals-19-01549-s001.zip › Figure-S16-Mn-C-TPP-ascorbate-synergy-Loewe-model.pdf]

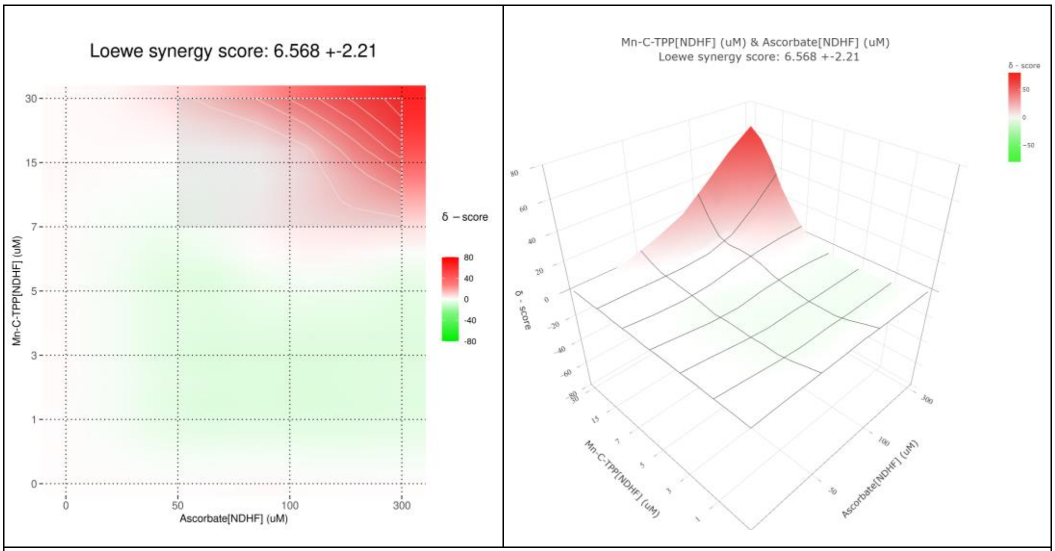
Loewe synergy score: 6.568 +-2.21
Mn-C-TPP[NDHF] (uM)
Ascorbate[NDHF] (uM)
δ − score
Mn-C-TPP[NDHF] (uM) & Ascorbate[NDHF] (uM)
Loewe synergy score: 6.568 +-2.21

(**a**)

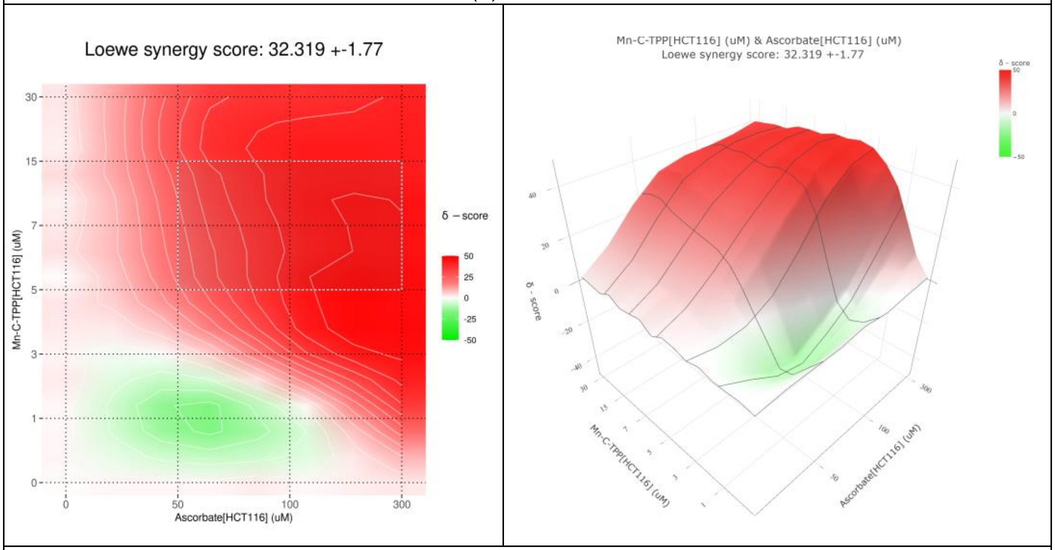
Loewe synergy score: 32.319 +-1.77
Mn-C-TPP[HCT116] (uM)
Ascorbate[HCT116] (uM)
δ − score
Mn-C-TPP[HCT116] (uM) & Ascorbate[HCT116] (uM)
Loewe synergy score: 32.319 +-1.77

(**b**)

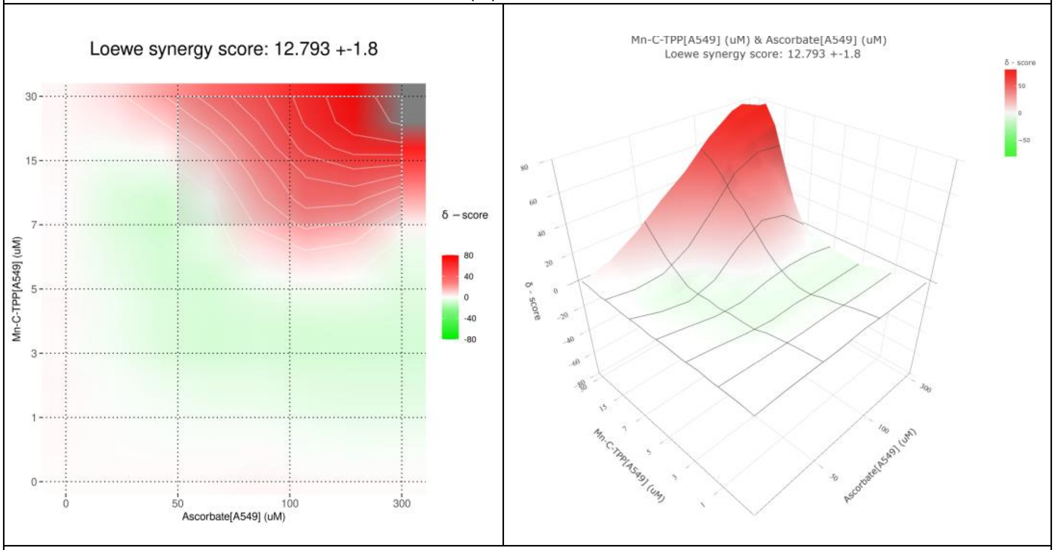
Loewe synergy score: 12.793 +-1.8
Mn-C-TPP[A549] (uM)
Ascorbate[A549] (uM)
δ − score
Mn-C-TPP[A549] (uM) & Ascorbate[A549] (uM)
Loewe synergy score: 12.793 +-1.8

(**c**)

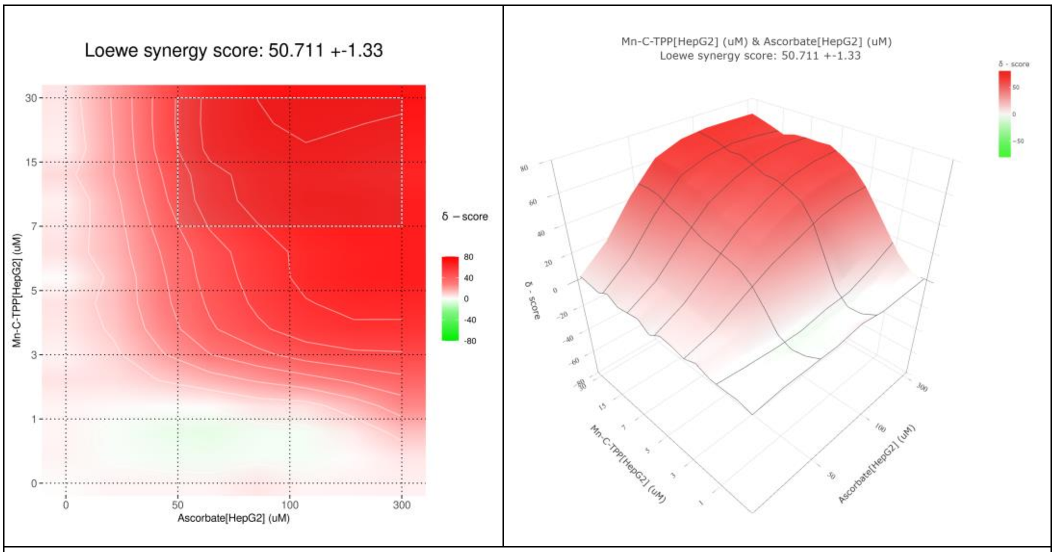


(**d**)

**Figure S16.** Two-dimensional (2D) synergy maps (left panels) and three-dimensional (3D) synergy surfaces (right panels) for the Mn–C–TPP and ascorbate combinations in: (**a**) NDHF, (**b**) HCT116, (**c**) A549, and (**d**) HepG2 cells following 72-hour exposure, generated with SynergyFinder 3.0 by using the Loewe model. The Loewe summary synergy scores are reported with the ± 95% confidence interval. Color scales represent Loewe synergy score ranges. The scores were NDHF: 6.568 ± 2.21, HCT116: 32.319 ± 1.77, A549: 12.793 ± 1.8, and HepG2: 50.711 ± 1.33.
